# PKD-YOLOv8: A Collaborative Pruning and Knowledge Distillation Framework for Lightweight Rapeseed Pest Detection

**DOI:** 10.3390/s25165004

**Published:** 2025-08-13

**Authors:** Haifeng Yu, Qingting Luo, Wei Peng, Lingyi Zheng, Jingjing Ju, Hui Zhuo

**Affiliations:** College of Information and Intelligence, Hunan Agricultural University, Changsha 410128, China; yuhaifeng@stu.hunau.edu.cn (H.Y.); luoqingting20@163.com (Q.L.); 973019230@stu.hunau.edu.cn (W.P.); zhenglingyi@stu.hunau.edu.cn (L.Z.); jujingjing1998@163.com (J.J.)

**Keywords:** oilseed rape pest detection, YOLOv8, model pruning, knowledge distillation

## Abstract

As an important oil and vegetable crop, rapeseed is widely planted and has important economic value worldwide. Rapeseed is often threatened by various pests during its growth. In order to effectively deal with rapeseed pests, this paper proposes a lightweight method based on collaborative compression learning. This method uses YOLOv8s as the basic model, combines model structure analysis and pruning sensitivity evaluation, and implements structured pruning to compress the model size. The Logit distillation method is integrated with the improved generative distillation method MGD, and the LMGD distillation strategy is proposed to enhance the student model’s ability to fit the teacher model’s feature expression. In order to verify the effectiveness of the proposed method, we built a rapeseed pest dataset (ACEFP) and conducted experiments. The improved model achieved 96.7% mAP@0.5, 93.2% accuracy, and 92.7% recall, while the parameter size was compressed from 11.2 MB to 4.4 MB, and the FLOPs were reduced from 28.3 G to 10.01 G, which were reduced by about 60.7% and 64.6%, respectively, and the accuracy was only reduced by 0.1%. The model achieved a measured frame rate of 11.76 FPS on the Jetson Nano edge device, demonstrating excellent real-time inference performance.

## 1. Introduction

Rapeseed, one of China’s major economic crops, is frequently attacked by pests such as aphids, cabbage worms, stink bugs, flea beetles, and leaf beetles during its growth cycle [1]. These pests not only lead to reduced yields but also seriously threaten crop quality and economic benefits. Traditional prevention and control methods rely mainly on experience and routine observation, which are subject to information lags and untimely responses. Therefore, the development and application of rapeseed pest and disease monitoring and early warning technologies are of great significance. With the development of deep learning technology, automated detection methods based on computer vision have gradually become a research hotspot.

In recent years, deep learning-based target detection technology has shown significant potential in agricultural pest identification and has become a research hotspot for smart agricultural management. For example, Gan et al. [2] proposed an improved YOLOv8-DBW model for corn leaf pest detection, which significantly improved detection accuracy and model lightweightness, making it suitable for mobile deployment. Zhao et al. [3] combined YOLOv5 with drone imagery to achieve accurate identification of rice field canopy pests and diseases, verifying the practicality of deep models in field scenarios. Xie et al. [4] designed the SEDCN-YOLOv8 model for cucumber pest detection in complex natural backgrounds, introducing deformation convolution and attention mechanisms to enhance the model’s robustness to small targets and occlusions. These achievements reflect the widespread application of target detection in crop pest and disease identification.

The existing mainstream target detection methods can be divided into two categories: two-stage and single-stage. Two-stage methods such as R-CNN [5] and Faster R-CNN [6] achieve high accuracy by first generating candidate regions and then performing classification and regression, but the computational complexity is high; single-stage methods such as SSD [7] and the YOLO series [8] directly locate and identify targets in an end-to-end manner. With the advantages of simple structure, fast inference speed, and convenient deployment, they are more suitable for resource-constrained agricultural automation scenarios. Since its introduction in 2016, the YOLO series has been continuously iterated, gradually optimizing the balance between detection accuracy, speed, and robustness. The YOLOv8 [9] architecture is well-known for its real-time target detection performance. It uses the CSPDarknet53 backbone network with a C2f module, which replaces the traditional CSPLayer to improve feature extraction and small target detection capabilities. Its Neck combines the Spatial Pyramid Pooling Fast (SPPF) layer, which accelerates calculations through fixed-size feature maps to achieve efficient multi-scale detection. The decoupled Head design handles target, classification, and regression tasks separately, thereby improving detection accuracy. However, YOLOv8 still has problems such as a large number of parameters and high computational cost, and its deployment in edge devices or embedded systems faces challenges, especially in meeting the real-time and low-power requirements in agricultural scenarios.

In response to the above bottlenecks, model compression technology has become a key research direction for improving the deployability of detection models. Its core is to reduce model complexity through strategies such as lightweight design, pruning, quantization, and knowledge distillation. Among them, pruning removes redundant structures by evaluating the importance of parameters, such as weight-based unstructured pruning [10] and structured pruning based on BN channel scaling factors [11]. Knowledge distillation improves the performance of lightweight student models by transferring the knowledge of large teacher models to them. The soft label distillation proposed by Hinton et al. [12] laid the theoretical foundation. Zagoruyko et al. [13] strengthened the transfer of intermediate features through the attention mechanism. Park et al. [14] further improved the generalization ability by using the knowledge of the relationship between samples.

Existing studies have verified the effectiveness of these technologies in agricultural pest detection. For example, Wang B et al. [15] combined hyperspectral imaging with 3D convolution to suppress noise and capture spectral–spatial features; Xiao Z et al. [16] constructed a lightweight network through one-dimensional convolution and attention mechanism; Kuzuhara et al. [17] designed a two-stage detection framework (YOLOv8 region proposal + Xception re-identification); Ullah et al. [18] developed DeepPestNet to improve generalization through data augmentation. However, these works mostly focus on a single compression strategy, making it difficult to achieve the optimal trade-off between model compactness and detection performance, especially in complex multi-target scenarios.

To this end, this study uses YOLOv8s as a baseline model and introduces targeted enhancements to better suit the needs of field detection scenarios, particularly in terms of detection performance and deployment efficiency. Specifically, we calculate the mean gamma coefficient of all BatchNorm layers in the YOLOv8s architecture and use this as a pruning sensitivity metric to perform global pruning on the model, resulting in a lighter, more compact model. Furthermore, to address the difficulty of traditional distillation methods in adequately representing sparse features after pruning, this paper proposes a collaborative distillation strategy, LMGD, that combines logit distillation with improved generative distillation. Logit distillation uses a single detection head for distillation between the compact model and the teacher model, leveraging high-level semantic and decision-making information to assist in evaluating the feature extraction quality of the student model. Improved generative distillation helps the student model learn the structure and representation patterns of the data abstracted by the teacher model, thereby improving its understanding of details and local structure. Combined, the compact model acquires more comprehensive knowledge. The main contributions of this work are as follows:

1. A compression framework combining channel pruning and dual distillation strategies is designed to improve inference speed while maintaining accuracy.
2. Developed a rapeseed pest image dataset covering five typical pest categories to support model training and evaluation.
3. Comprehensive comparative experiments demonstrated the effectiveness of the proposed model in balancing detection performance and deployment efficiency. While achieving 96.7% mAP@0.5, 93.2% precision, and 92.7% recall, the model was able to compress the parameter size from 11.2 MB to 4.4 MB and the FLOPs from 28.3 GB to 10.01 GB, representing reductions of approximately 60.7% and 64.6%, respectively. This approach improved model inference efficiency while only decreasing detection accuracy by 0.1%. The improved model achieved a measured frame rate of 11.76 FPS on a Jetson Nano edge device, demonstrating excellent real-time inference performance.

## 2. Related Work

### 2.1. YOLO Model

YOLO (You Only Look Once) is the first version of the YOLO series of object detection algorithms, proposed by Joseph Redmon et al. [8] in 2016. YOLO’s core idea is to transform the object detection problem into a regression problem, allowing for simultaneous prediction of both the object’s class and location through a single forward pass. YOLO’s core concept is to divide the input image into an S×S grid, with each grid responsible for predicting a certain number of bounding boxes and class probabilities. YOLO completes detection with a single forward pass, whereas traditional object detection algorithms (such as R-CNN) typically require multiple steps of region proposal and classification. YOLO is an end-to-end model that directly outputs detection results from the input image, without the complex region proposal step. Despite the success of YOLO and its subsequent related work, its high computational cost has limited its practical application [19,20]. Many researchers are exploring more efficient model compression methods to promote the widespread application of YOLO in real-world scenarios.

### 2.2. Structured Pruning

As a mainstream model compression method, channel pruning can systematically remove redundant channels in convolutional neural networks, thereby effectively reducing the number of model parameters, computational complexity, and memory usage and improving inference speed. Research on channel pruning mainly focuses on designing channel importance evaluation methods. Theis et al. [21] quantified the sensitivity of channels to changes in model parameters through the Fisher information matrix and measured the gradient contribution of channels to the loss function. He et al. [22], based on the principle of minimizing Euclidean space distance, located and filtered redundant channels in multi-branch networks by taking advantage of the strong robustness of geometric median outliers. Lin et al. [23] used singular value decomposition to analyze the linear independence of the channel matrix. The lower the rank, the higher the redundancy of the channel group. Based on this, a global importance score was constructed to achieve information retention at a high compression rate. As the finest-grained structured pruning method, channel pruning also causes relatively little performance damage to the model. However, when the pruning rate is too high, relying solely on pruning methods will inevitably lead to information loss.

### 2.3. Knowledge Distillation

In order to retain more feature information during the model compression process, the reasoning performance can be improved through knowledge distillation technology. Knowledge distillation is a model compression technology that aims to transfer the “knowledge” contained in the large teacher model to the small student model to improve the reasoning performance and generalization ability of the small model while reducing computational overhead. The knowledge distillation model first proposed by Hinton et al. [12] is based on network output knowledge distillation. This distillation model achieves distillation from the teacher network to the student network by minimizing the distribution difference between the output layers of the teacher network and the student network. Chen et al. [24] first designed a classification-regression dual-branch distillation framework for the target detection task and achieved positioning knowledge transfer through bounding box relationship loss. Zhang et al. [25] proposed a hierarchical distillation strategy to jointly optimize the backbone features and key point response distribution in the real-time pose estimation task. Romero et al. [26] pioneered the intermediate layer feature imitation paradigm, using the adapter layer to map the deep features of the teacher to the student network to solve the problem of model depth mismatch. Zagoruyko et al. [13] introduced an attention transfer mechanism, generating a spatial heat map based on the channel absolute mean to guide students to focus on key areas, significantly reducing the false positive rate of target detection. Lee et al. [27] proposed an SVD self-distillation method, extracting the principal components of features through singular value decomposition to filter out redundant noise. Passban et al. [28] designed a dynamic layer projection mechanism (ALP-KD) to align the inter-layer knowledge of heterogeneous teacher and student models using a learnable projection matrix.

## 3. Materials and Methods

### 3.1. Dataset

To construct a high-quality dataset for rapeseed pest identification, this study collected an image dataset, ACEFP (Annotated Cabbage-field Efficient Pest Dataset, v.1.8.6), containing five common pest categories. This dataset was collected at the Yunyuan Rapeseed Base in Changsha, China (longitude: 113.083204, latitude: 28.180986). Images were manually captured using a Nikon D3500 (Nikon Corporation, Tokyo, Japan) to ensure sample authenticity and environmental diversity. The pests included are aphids, cabbage worms, cabbage stink bugs, flea beetles, and monkey leaf beetles. A total of 1010 valid images were collected, with the following class distribution: 220 aphids, 190 cabbage worms, 185 cabbage stink bugs, 210 flea beetles, and 205 monkey leaf beetles. All images were manually annotated using the LabelImg 1.8.6 tool, with reference images provided to ensure accurate class annotations. The images were saved in the YOLO format for downstream detection model training and evaluation.

**Figure 1:**
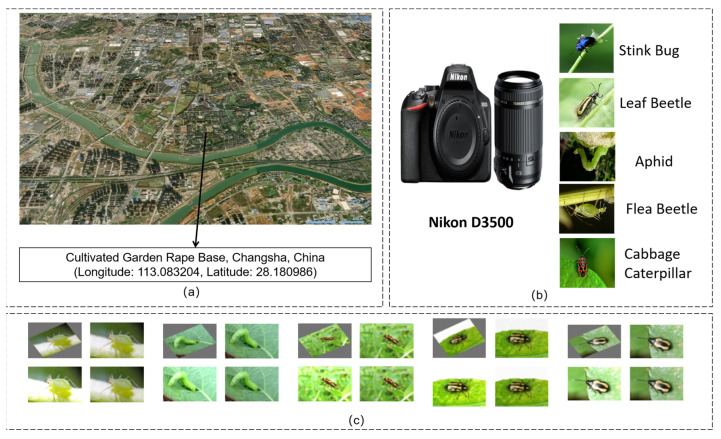
(**a**) Place of collection of datasets. (**b**) Data capture equipment. (**c**) Data enhancement.

To address complex field environmental disturbances (such as foliage obstruction and uneven lighting), we employed a series of random data augmentation strategies to improve model generalization, as shown in Figure 1. Specifically, these strategies include artificially adding Gaussian noise during training to simulate sensor noise and environmental interference, enhancing the model’s robustness to noise; applying geometric transformations such as rotation (±30°), translation (±20%), and scaling (0.8–1.2×) through coordinate system transformation to simulate the diversity of target pose, position, and scale, improving the model’s robustness to such geometric variations; and implementing brightness adjustment (±30%) to simulate the variable lighting conditions found in the field, enhancing the model’s illumination invariance, as shown in Figure 1. The augmented dataset is divided into a training set (2121 images), a validation set (606 images), and a test set (303 images) in a 7:2:1 ratio. The specific distribution is shown in Table 1.

### 3.2. Method

#### 3.2.1. Overview

Unlike traditional single-path optimization approaches of either independent pruning or distillation, this study analyzes the changes in feature distribution before and after pruning and proposes a comprehensive optimization strategy, PKD, that integrates pruning and distillation. Innovations include 1. Improved distillation mechanism: To address the reduced number of feature channels after pruning, this approach abandons the traditional forced alignment of feature maps and directly feeds the sparse features of the student model into the generative module (3 × 3 double-layer convolution), achieving local-to-global semantic alignment; 2. Dual supervision: Improved generative distillation is used in the feature space to enhance the expressive power of intermediate features, while Logit distillation of the teacher detection head constrains the output distribution in the output space. This approach significantly reduces computational complexity while maintaining detection accuracy, resulting in a lightweight model that combines efficiency and reliability.

#### 3.2.2. YOLOv8 Benchmark Model Architecture

**Figure 2:**
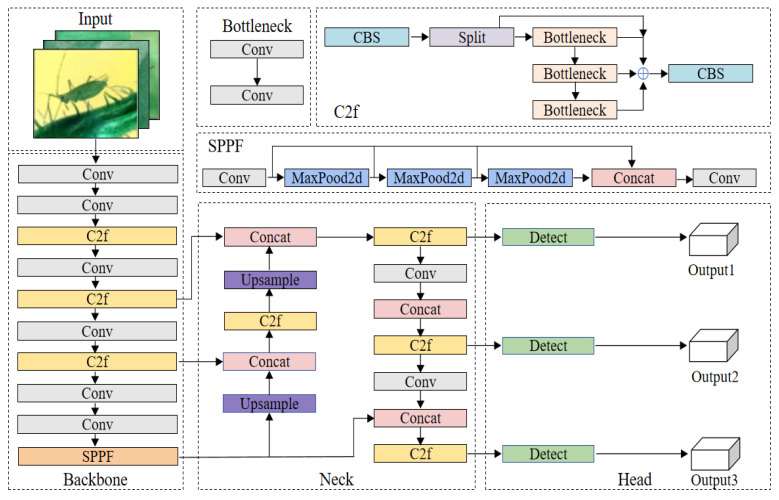
Diagram of the YOLOv8 network.

As the representative version of the YOLO series, YOLOv8 inherits the advantages of real-time detection while achieving breakthroughs in multi-task performance. Through systematic architectural innovations, this model demonstrates an excellent precision-speed balance in tasks such as object detection and instance segmentation. Its network structure consists of three parts: the Backbone adopts an enhanced CSPDarknet53 architecture, which works in conjunction with the SPPF layer through cross-stage dense connections to effectively extract local image features and achieve multi-scale spatial information fusion; the Neck integrates the PAA module and a dual-path PANet, constructing a scale-invariant feature space through a bidirectional feature pyramid to enhance cross-scale object detection capabilities; the Head adopts a decoupled detection head design, combined with an anchor-free mechanism and task alignment strategy, significantly optimizing localization accuracy and classification confidence in dense scenarios. Ultimately, it achieves a high-precision and speed balance in real-time inference, providing a reliable technical foundation for real-time agricultural monitoring. Figure 2 shows the YOLOv8 model structure.

#### 3.2.3. Channel Pruning Based on γ Coefficients

Channel pruning first uses specific mathematical criteria to measure the importance of the channel, discarding the channels with low scores to achieve a light weight. In convolutional neural networks, the BN layer accelerates the convergence of the neural network and improves the generalization ability by reducing the internal covariate shift during training. Its core formula is

(1) $$\hat{Z}=\frac{z-\mu }{\sqrt{\sigma^{2}+\varepsilon }};\;y=\gamma \;\hat{z}+\beta$$

Here, *z* is the output feature of the convolutional layer, μ and $\sigma^{2}$ are the mean and variance of the current batch of data, respectively, ε is the numerical stability coefficient, and γ and β are learnable scaling and translation factors. γ controls the reconstruction amplitude of the normalized features, giving the network the ability to autonomously adjust the distribution of normalized features. Specifically, the model repeatedly iterates the γ parameter during continuous training. When the network training converges, if the γ value of a channel is greater than 1, it is considered a critical channel. Conversely, if it approaches 0, it indicates that the channel is redundant and the output degenerates to the constant β, losing its nonlinear expression ability. At this point, it can be safely pruned.

Therefore, the channel importance score $S_{c}$ can be set according to the parameter to perform global channel importance evaluation. The specific formula is as follows:

(2) $$S_{c}=\frac{\left|\gamma_{c}\right|}{\sum_{i=1}^{C}\left|\gamma_{i}\right|}$$

where *C* is the total number of channels. $\gamma_{c}$ represents the BN γ coefficient of channel *c*. $S_{c}$ represents the ratio of the sum of the absolute values of the channel parameters to the total absolute values of all channels. The smaller the value, the less important the channel is and the more likely it is to be pruned.

At the same time, in order to further guide channel sparsity, the L1 regularization term can be introduced into the total loss function to compress the γ value of unimportant channels. The specific formula is as follows:

(3) $$L_{total}=L_{task}+\lambda \sum_{l=1}^{L}\sum_{c=1}^{C^{\left(l\right)}}\left|\gamma_{c}^{\left(l\right)}\right|$$

where *C* is the total number of channels. $L_{task}$ is the task loss (such as cross entropy loss), λ is the regularization strength coefficient, and $C^{\left(l\right)}$ is the number of channels in the lth layer. L1 regularization tends to push the γ parameters of unimportant channels toward 0 while retaining the γ values of important channels. At the same time, the degree of sparsity can be controlled by adjustment the λ to prevent excessive sparsity from causing precision loss.

**Figure 3:**
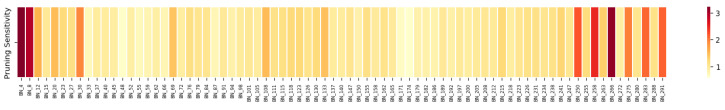
Layer-wise Pruning Sensitivity of YOLOv8 Based on BN γ Coefficients.

To better understand the differences in sensitivity of YOLOv8 layers to channel pruning, this paper calculated and visualized the mean gamma coefficients of all BatchNorm layers, constructing a pruning sensitivity heatmap as shown in Figure 3. The horizontal axis represents the index position of each BatchNorm layer in the model, while the vertical axis represents the pruning sensitivity index, with the color depth reflecting the magnitude of the gamma coefficient. The results show that the early and late stages of the model (such as BN_4 and BN_266) have significantly high sensitivity regions, suggesting that their feature expression remains highly dependent on channels, while intermediate layers (such as BN_80 to BN_160) generally exhibit low sensitivity, indicating the presence of redundancy that can be pruned.

**Figure 4:**
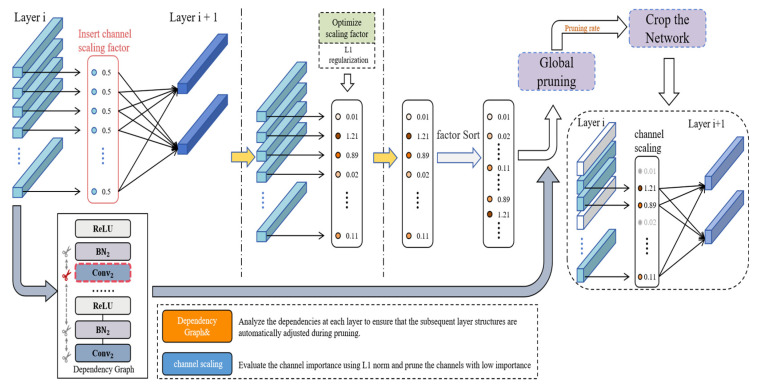
Pruning flowchart.

Based on this, this paper proposes a global channel pruning strategy based on sensitivity guidance: using the BN γ coefficient as a unified importance metric, a more aggressive pruning ratio is imposed on low-sensitivity layers to maximize the compression benefit; while a conservative strategy is adopted for high-sensitivity layers to reduce their structural perturbations, thereby achieving refined and non-uniform cross-layer compression. During the training phase, an L1 regularization constraint [29] is introduced to induce channel sparsity. After training, a global threshold is set based on the γ value for pruning operations to avoid post-processing dependence and ensure the simplicity of the model structure and the robustness of performance. The pruning process is shown in Figure 4.

#### 3.2.4. Collaborative Distillation Strategy LMGD

To address the problem of traditional distillation methods struggling to adequately represent sparse features after pruning, this paper proposes a collaborative distillation strategy, LMGD (Logit and Modified Generative Distillation), that combines logit and modified generative distillation. This approach combines two supervisory signals to enhance the student model’s feature representation and decision accuracy.

**Figure 5:**
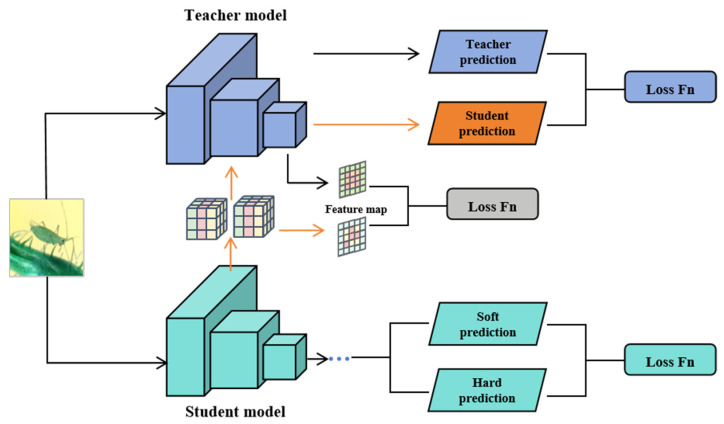
LMGD frame diagram.

**Figure 6:**
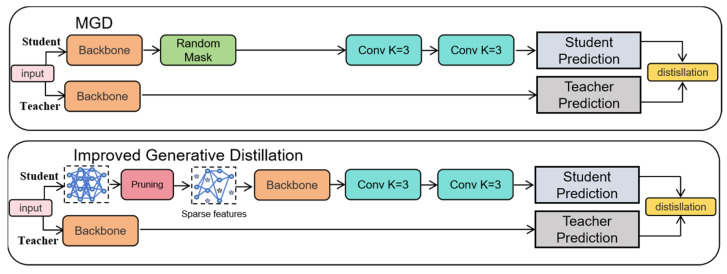
Improved Raw Distillation vs. MGD Methods.

As shown in Figure 5, the overall structure of the LMGD framework includes an improved generative distillation module and a logit distillation mechanism. To clearly illustrate the difference between the proposed method and traditional generative distillation (MGD), Figure 6 compares the two.

Compared to the traditional MGD method (Figure 6, top), the improved generative distillation module proposed in this paper (Figure 6, bottom) eliminates redundant masking operations and directly uses pruned sparse features as generator input, focusing more on the feature reconstruction process. This improves the alignment between pruned features and teacher features, as well as reconstruction efficiency.

LMGD also introduces a logit distillation mechanism. By minimizing the difference in probability distributions between the student and teacher models at the category prediction layer, it strengthens the student model’s ability to learn the teacher’s discriminative boundaries, thereby improving overall inference accuracy. Furthermore, the student model’s feature maps are input to the teacher model’s detection head, further ensuring high semantic consistency in the output predictions.

To enhance the expressive power and computational efficiency of the reconstruction module, this paper designs a lightweight feature generation module consisting of two 3 × 3 convolutional layers. Compared to a single large-kernel convolutional structure, this module reduces the number of parameters and computational cost while maintaining an effective receptive field. The first convolution layer is responsible for local feature extraction, while the second convolution layer integrates semantic information to achieve high-quality reconstruction of the pruned feature map, providing a stable intermediate representation for the distillation process.

The LMGD method uses both generative distillation and logit distillation for dual optimization, strengthening feature alignment and decision output, respectively, significantly improving detection accuracy and reducing computational complexity. Training losses include bounding box regression, confidence loss, category loss, and distillation loss. The latter is composed of generative distillation and logit distillation losses. Hyperparameter weighting balances these losses to achieve fine-grained optimization. Loss calculation is as follows:

The loss calculation of improved generative distillation is as follows:

(4) $$L_{f}=\frac{1}{N}\sum_{i=1}^{N}\left|\right|G\left(F_{s}^{\left(i\right)}\right)-F_{t}^{\left(i\right)}{\left|\right|}_{2}^{2}$$

where $F_{s}^{\left(i\right)}$ and $F_{t}^{\left(i\right)}$ represent the feature maps of the student model and the teacher model at the *i*-th layer, respectively, and *G*(.) denotes the 3 × 3 convolutional generative module.

The loss calculation for Logit distillation is as follows:

(5) p=T_H(f)

(6) $$L_{l}=\frac{1}{B}\sum_{b=1}^{B}{\left\|p_{b}-{t\_p}_{b}\right\|}^{2}$$

where *f* is the feature map obtained after the student model passes through the generation module, *t_p* is the output of the teacher model, and *T_H* is the detection head of the teacher model. *p* is the output of the feature map of the student model after passing through the detection head of the teacher model, $p_{b}$ and ${t\_p}_{b}$ denote the outputs of the student model and the teacher model at the *b*th sample, and *B* denotes the batch size.

The total loss of the overall architecture is calculated as follows:

(7) $$L=\alpha_{1}\left(L_{bbox}+L_{conf}+L_{cls}\right)+\alpha_{2}L_{f}+\alpha_{3}L_{l}$$

where $L_{bbox}$, $L_{conf}$, and $L_{cls}$ denote the bounding box regression loss, confidence loss, and category loss, respectively, and $\alpha_{1}$, $\alpha_{2}$, and $\alpha_{3}$ are weight hyperparameters used to balance the contribution of different loss terms.

## 4. Experimental Results and Analysis

### 4.1. Experimental Configuration and Assessment Metrics

The experiments were conducted on a Windows 11 system using an Intel i7-13700KF CPU and an NVIDIA RTX 4090 GPU with 128 GB of system memory. The model was implemented using the PyTorch 2.5.1 framework and Python 3.8. The model was trained from scratch for 200 epochs with a 224 × 224 input resolution and a batch size of 32. A fixed random seed was used to ensure reproducibility. Adam was used as the optimizer with an initial learning rate of 2 × 10^−5^. Data loading was accelerated by adjusting the number of CPU threads, while all other parameters remained default. Pruning was performed using the pruning-torch tool, and no pretrained weights were loaded.

Performance evaluation covered both model lightweighting and detection accuracy. The pruning phase focused on parameter count, FLOPs, and mAP@0.5 as core metrics. The distillation phase further incorporated mAP@0.5:0.95, recall, and precision. Among them, AP calculates the average precision under different recall rates, reflecting the model’s comprehensive recognition ability for positive classes; mAP evaluates the robustness of the model by calculating the average precision under different IoU thresholds (0.5–0.95); the recall rate reflects the missed detection rate, the precision rate measures the false detection rate, and *TP*, *FP*, and *FN* represent the number of pests correctly detected, falsely detected, and missed, respectively.

The above indicator equation is as follows:

(8) $$Precison=\frac{TP}{TP+FP}$$

(9) $$Recall=\frac{TP}{TP+FN}$$

(10) $$AP=\int_{0}^{1}p(r)dr$$

(11) $$mAP=\frac{1}{m}\sum_{i=1}^{m}AP_{i}$$

### 4.2. Main Results

**Figure 7:**
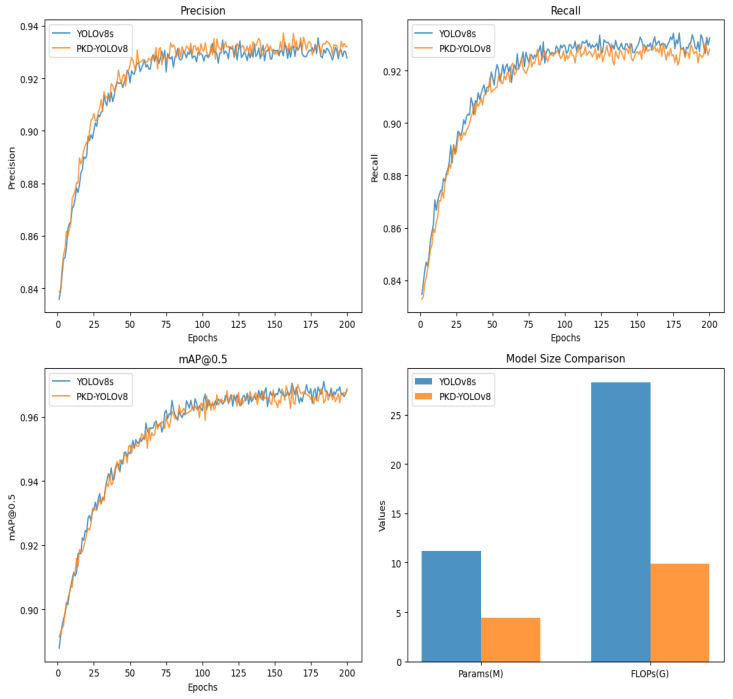
Results comparison of YOLOv8s and PKD-YOLOv8.

In this section, we conduct extensive and exhaustive experiments on the rapeseed pest image dataset, ACEFP, to validate the feasibility and superiority of our approach. The experimental results are shown in Figure 7. Figure 7 shows a comparison between the baseline YOLOv8s model and the improved PKD-YOLOv8 model. Precision, recall, mAP@0.5, parameters, and FLOPs are compared. The figure shows that the P and R values of both models exhibit significant fluctuations during the first 110 epochs. After the next 90 epochs, the curves gradually stabilize, with the final P and R values of the PKD-YOLOv8 model reaching 93.2% and 92.7%, respectively. For the mAP@0.5 metric, the curve fluctuates significantly during the first 130 epochs, but gradually stabilizes over the next 70 epochs. A comparison between the PKD-YOLOv8 and YOLOv8s models is also presented, focusing on the number of parameters and FLOPs. The optimized PKD-YOLOv8 model shows a 60.7% reduction in parameters and a 64.6% reduction in FLOPs. Overall, the PKD-YOLOv8 model achieves 96.7% mAP@0.5 while significantly reducing the overall model size. This is only a 0.1% decrease from YOLOv8s’ 96.8%, approaching the baseline performance and demonstrating further optimization.

To further demonstrate the effectiveness of our approach, we evaluated seven object detection models—YOLOv7, YOLOv5m, YOLOv5s, YOLOv8s, SSD, Faster-RCNN, and the proposed PKD-YOLOv8—under the same training conditions and datasets. The experimental results are shown in Table 2. Specifically, among the traditional YOLO models, YOLOv7 and YOLOv5m achieved mAP@0.5:0.95 values of 51.2% and 45.4%, respectively. However, these models suffer from high computational complexity of 105.6 G FLOPs and 48.2 G FLOPs, respectively, and parameter sizes of 36.7 M and 21.2 M, respectively. Lightweight alternatives, such as YOLOv5s, significantly reduce model complexity to 15.8 G FLOPs and only 7.2 M parameters. However, this reduction comes at the expense of lower detection accuracy, with a mAP@0.5:0.95 of 37.4%. In comparison, the baseline model YOLOv8s has 28.6 G FLOPs and 11.2 M parameters, achieving a detection accuracy of 58.6%, showing a better basic performance balance.

To further validate our approach, we also evaluated the classic SSD [7] and Faster R-CNN [30] models. SSD achieved a mAP of 49.3% with a computational overhead of 16.6 G FLOPs and 26.29 M parameters, respectively. Although Faster R-CNN achieved a relatively high mAP of 54.3%, its computational complexity and parameter count were substantial, at 370.2 G FLOPs and 137.10 M.

**Figure 8:**
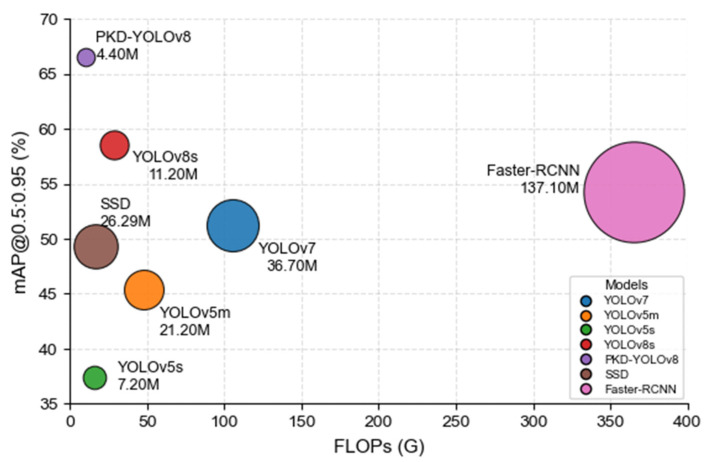
Accuracy–Speed Trade-off with Model Scale.

Nevertheless, as shown in Figure 8, all baseline models achieve lower accuracy than our proposed PKD-YOLOv8 model, which achieves 66.5% mAP@0.5:0.95. Notably, compared to YOLOv8s, its parameter count and FLOPs are reduced by 60.7% and 64.6%, respectively, highlighting its potential for real-time pest detection in resource-limited agricultural environments.

### 4.3. Visual Presentation of the PKD Framework

**Figure 9:**
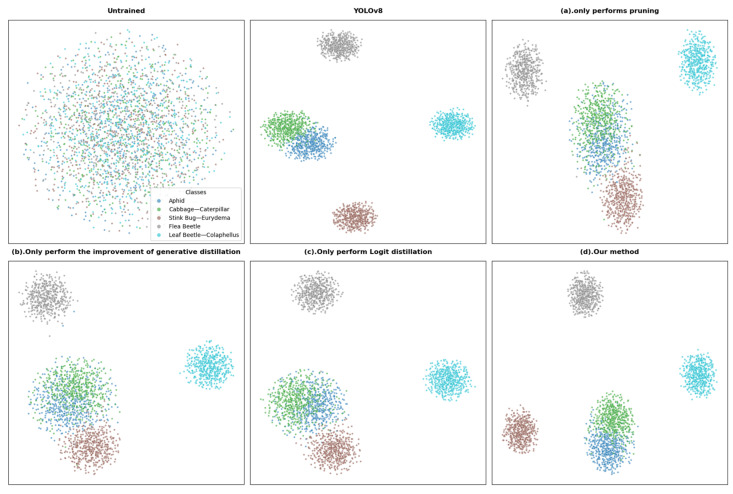
Feature visualization map.

To further demonstrate the effectiveness and interpretability of our method, we used t-SNE to perform feature extraction, dimensionality reduction, and visualization on the original data and the trained model features. This method extracted key features from the original data, reduced the data dimensionality, and achieved effective data visualization. The results are shown in Figure 9.

The figure shows the spatial distribution of features across different models on the target pest classification task: The untrained data distribution is mixed and disordered; the YOLOv8 baseline model forms clearly defined clusters for each pest, demonstrating excellent classification performance. Figure 9a (pruning only) shows blurred cluster boundaries and overlapping categories, indicating that pruning compromises representational capabilities. Figure 9b (improved generative distillation only) shows a distribution close to the baseline, but with increased cluster radius and the presence of outliers, indicating that it preserves structural information but lacks fine-grained discrimination. Figure 9c (logit distillation only) forms clear clusters but exhibits unusually compact regions, reflecting overconfidence caused by its reliance on soft labels. Figure 9d (our approach) achieves the best overall performance, achieving clustering close to the baseline and exhibiting superior inter-class spacing. Its effectiveness is primarily attributed to the following:

Logit distillation utilizes a shared detection head between the student and teacher models, leveraging high-level semantic and decision-making information to aid in evaluating the student model’s feature extraction quality. Improved generative distillation can help the student model learn the structure and representation patterns of the data abstracted by the teacher model, thereby improving the model’s understanding of details and local structures. When combined, the compact model can acquire more comprehensive knowledge.

### 4.4. Ablation Study

To comprehensively evaluate the performance of the model, we adopt YOLOv8 as the baseline model and conduct a series of detailed ablation experiments on the effectiveness of each module, the determination of the pruning ratio, and the selection of the knowledge distillation strategy.

#### 4.4.1. Effectiveness of Each Module

We selectively added or removed specific modules from the baseline model for comparison. As shown in Table 3, the accuracy of the pruned model alone (Group A) dropped significantly to 95.6% due to the lack of knowledge transfer. After introducing improved generative distillation (Group B), mAP@0.5 was restored to 96.3% by reconstructing the multi-scale features of the teacher network. Logit distillation (Group C), leveraging the semantic supervision of the teacher detection head, improved accuracy to 96.4%. When the two were optimized together (Group D), the dual feature-output supervision mechanism achieved mAP@0.5 of 96.7%, approaching the 96.8% of the original teacher model. This demonstrates that improved generative distillation suppresses the loss of fine-grained information caused by pruning by enhancing local structure perception, while logit distillation transfers high-level semantic decision logic. The two complement each other to alleviate the problem of feature degradation.

#### 4.4.2. Model Performance with Different Pruning Ratios

The pruning ratio is a key hyperparameter in our method. Increasing the pruning ratio significantly reduces the number of model parameters, but also increases the likelihood of incorrectly pruning key channels, affecting the final prediction. To find the optimal pruning ratio, we conducted a series of experiments, the results of which are shown in Table 4. By comparing and analyzing model performance at different ratios, we ultimately selected 70% as the optimal pruning ratio.

#### 4.4.3. Model Performance Under Different Knowledge Distillation Strategies

**Figure 10:**
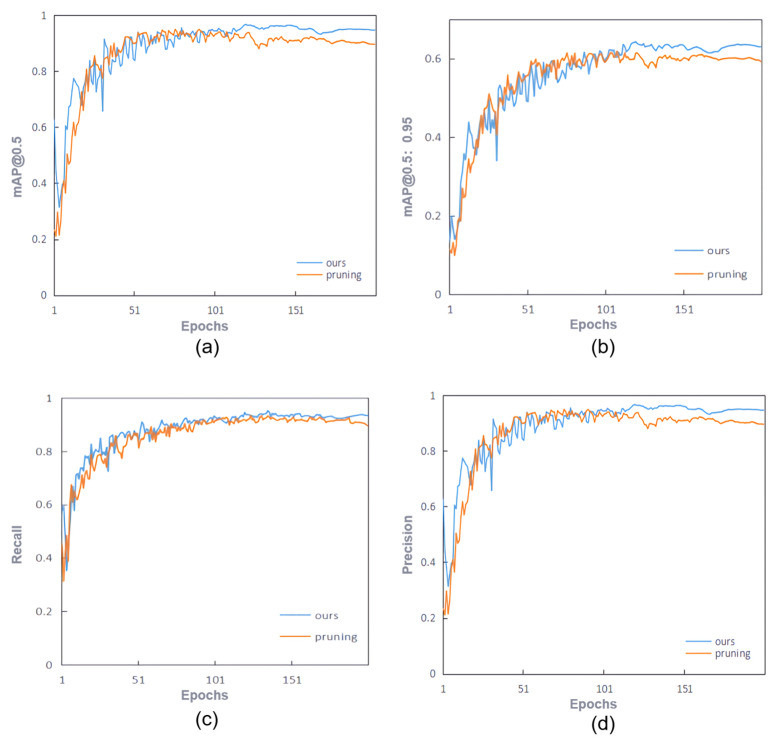
The comparison curve between the improved model and the compact model. (**a**) mAP@0.5; (**b**) mAP@0.5:0.95; (**c**) Recall; (**d**) Precision.

To validate the effectiveness of our method, we compared the performance of a model fine-tuned using the LMGD method with a compact model pruned at a 70% rate on four metrics: mAP@0.5, mAP@0.5:0.95, Recall, and Precision. The experimental results are shown in Figure 10. As shown in Figure 10, our method significantly outperforms the compact model in all four metrics. The two methods achieved similar detection accuracy in the first 100 training rounds, but as the number of training rounds increases, the LMGD method demonstrates superior performance, and the gap widens. This demonstrates that the LMGD method effectively prevents overfitting and consistently performs well on the test set.

To more comprehensively evaluate the effectiveness of this method, we conducted a series of experiments on the selection of knowledge distillation strategies for compact models and compared various classic distillation methods. The experimental results are shown in Table 5. The experimental results show that from the perspective of mAP@0.5, the detection accuracy of our method reaches 96.7%, which is 0.9%, 0.4%, 0.4%, 0.6%, and 1.0% higher than the KD [31], MGD [32], Cross KD [33], CWD [34], and FitNet [26] methods, respectively. While maintaining a 70% reduction in model parameters, the accuracy only decreases by 0.1% compared to the teacher model before pruning and improves by 1.1% compared to the student model after pruning. The detection accuracy of each category is at least 0.1% higher than the comparison method, and the maximum is only 0.2% lower.

**Figure 11:**
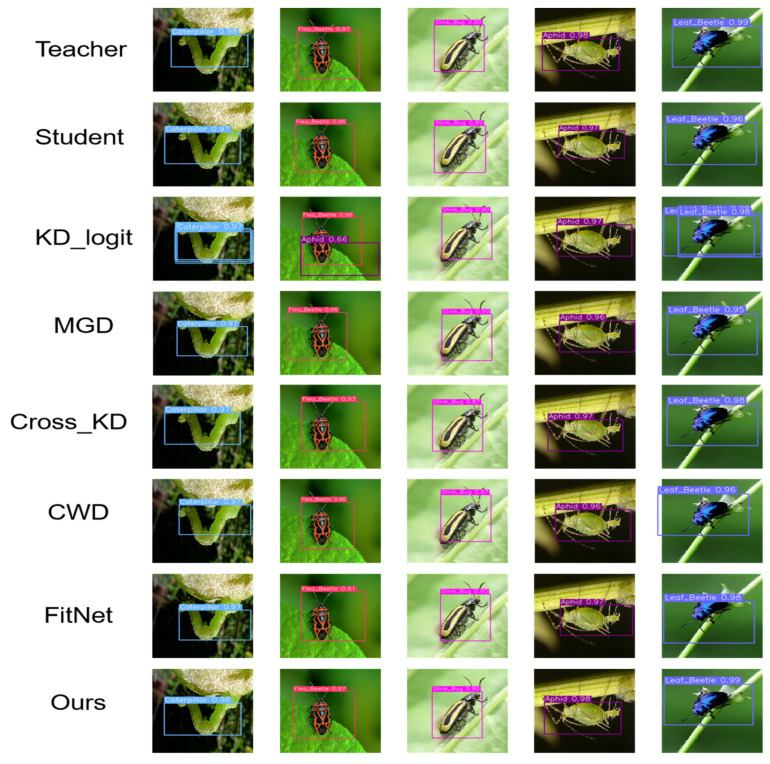
Comparison of the detection results of some validation sets.

To further demonstrate our method, we provide visualizations of the detection results for each knowledge distillation strategy. Figure 11 shows the visualization of different models for detecting five types of rapeseed pests. For aphids, while most models correctly identify the target, there are slight deviations between the Student and Cross KD models. Our method’s bounding boxes fit the insect body better and achieve higher confidence. For cabbage worm detection, the bounding boxes of traditional methods are slightly wider and easily overlap with the background. Our method’s bounding boxes tightly enclose the main body of the insect, resulting in more accurate detection. For stink bugs, flea beetles, and leaf beetles, methods like MGD and FitNet experience bounding box shifts due to complex backgrounds. However, our method maintains high accuracy, with bounding boxes highly consistent with the insect’s outline. Notably, when detecting samples with rapeseed pests, the KD method generates multiple anchor boxes, while other methods do not. This may be due to limitations in the KD method’s feature transfer process. Overall, our method demonstrates higher detection accuracy, bounding box accuracy, and prediction information reliability in the five pest detection tasks shown in Figure 11, further validating its detection performance and adaptability.

### 4.5. Model Deployment and Inference Performance Evaluation on Jetson Nano

To verify the deployability and real-time performance of the proposed lightweight approach in real-world edge computing scenarios, this study used the NVIDIA Jetson Nano B01(NVIDIA Corporation; Santa Clara, CA, USA) embedded platform as a test device to test and evaluate the model’s inference speed on a real-world device.

The Jetson Nano is an embedded development board designed specifically for edge computing and lightweight AI applications. Its compact size (100 × 80 × 29 mm) facilitates integration and deployment. The platform utilizes an NVIDIA Maxwell architecture GPU with 128 integrated CUDA cores, a quad-core ARM Cortex-A57 CPU (1.43 GHz), and 4 GB of built-in LPDDR4 video memory, supporting parallel inference and efficient data processing. Table 6 shows the detailed hardware configuration of the Jetson Nano.

To adapt the model to the Jetson Nano platform, the model must be converted from PyTorch to ONNX format. A deployment environment must then be established using the JetPack SDK, which includes TensorRT, CUDA, and cuDNN. TensorRT is then used to perform graph optimization and precision compression on the ONNX model to improve inference efficiency.

After deployment, we conducted unified inference tests on the original YOLOv8 model and the lightweight PKD-YOLOv8 model on the same test set, recording the average inference time and corresponding frame rate (FPS) for a single frame. All tests were performed in Jetson Nano high-performance mode. The results are shown in Table 7.

Test results show that the original YOLOv8 model’s average single-frame inference time on a Jetson Nano is 137 ms, with an FPS of 7.29. The PKD-YOLOv8 model, optimized through pruning and distillation, reduces this average inference time to 85 ms, with an FPS of 11.76, an improvement of approximately 38%. This enhances the model’s real-time inference capabilities on low-power edge devices. This result validates the effectiveness and practicality of this approach in real-world deployment scenarios.

## 5. Conclusions

This paper addresses the challenges of model deployment and low detection accuracy in rapeseed field pest identification. We constructed a field dataset containing five common pest types and proposed a collaborative compression learning method that combines model pruning and knowledge distillation. Based on structural analysis of the YOLOv8 model and layer sensitivity evaluation, we performed structured pruning and introduced an improved knowledge distillation strategy to improve the detection performance of the lightweight model. The effectiveness of the proposed method was verified through a series of experiments in a unified experimental environment. Our method achieved 96.7% mAP@0.5, 93.2% accuracy, and 92.7% recall, while reducing the parameter size from 11.2 MB to 4.4 MB and the FLOPs from 28.3 GB to 10.01 GB, representing reductions of approximately 60.7% and 64.6%, respectively. This method improved model inference efficiency while only decreasing detection accuracy by 0.1%. We hope that the proposed method will contribute to future performance improvements in the YOLO family. Despite this, this study still has certain limitations: data collection was concentrated in a specific region, resulting in relatively limited sample diversity; the model has not yet been deployed and tested on real agricultural equipment, and system integration and real-time performance require further verification.

Future work will focus on two aspects: first, promoting the deployment of the model in actual agricultural terminal devices, such as drones and mobile terminals, combining high-resolution image acquisition and wireless transmission to achieve efficient field pest monitoring; second, expanding data sources, continuously collecting pest images at different times, locations, and weather conditions, to improve the model’s generalization and robustness.

In addition, although the proposed method was developed based on YOLOv8, its core strategies—including pruning-aware analysis and collaborative compression with distillation—are model-agnostic and can be extended to other YOLO series models such as YOLOv9 to YOLOv12, which share similar architectural patterns. This enables the method to retain compatibility with other YOLO versions, increasing its practical utility.

## Figures and Tables

**Table 1:** Data enhancement chart.

Category	Original Data	Augmented Data	Training Set	Validation Set	Test Set
Aphid	150	650	455	130	65
Cabbage Caterpillar	130	563	394	113	56
Stink Bug–Eurydema	125	541	379	108	54
Flea Beetle	140	606	424	121	61
Leaf Beetle–Colaphellus	155	670	469	134	67
Total	700	3030	2121	606	303

**Table 2:** Multi-dimensional performance comparison of models.

Models	mAP@0.5:0.95 (%)	FLOPs (G)	Parameters (M)
YOLOv7	51.2	105.6	36.7
YOLOv5m	45.4	48.2	21.2
YOLOv5s	37.4	15.8	7.2
YOLOv8s	58.6	28.6	11.2
SSD	49.3	16.6	26.29
Faster-RCNN	54.3	370.2	137.10
PKD-YOLOv8	66.5	10.01	4.4

**Table 3:** Ablation experiment.

Models	Pruning	Feature KD	Logit KD	mAP@0.5 (%)
YOLOv8				96.8
Group A	√			95.6
Group B	√	√		96.3
Group C	√		√	96.4
Group D	√	√	√	96.7

**Table 4:** Performance metrics of model pruning.

Pruning Ratio	0%	10%	30%	70%	90%
model parameter (M)	11.2	10.0	8.3	4.4	2.6
FLOPs (G)	29.3	26.4	20.7	11.0	6.3
mAP@0.5 (%)	96.8	96.7	96.2	94.6	93.7

**Table 5:** Comparative experiment.

Method	mAP@0.5 (%)	Aphid (%)	Cabbage Caterpillar (%)	Stink Bug–Eurydema (%)	Flea Beetle (%)	Leaf Beetle–Colaphellus (%)
Teacher	96.8	98.2	98.2	90.9	97.4	99.2
Student	95.6	97.1	97.0	89.8	96.3	98.1
KD_logit	95.8	97.2	97.3	90.2	96.0	98.4
MGD	96.3	97.8	97.8	90.5	96.5	98.8
Cross KD	96.3	97.8	97.3	90.7	96.5	98.6
CWD	96.1	97.6	97.2	90.6	96.4	98.6
FitNet	95.7	97.3	97.1	90.1	96.1	98.2
LMGD	96.7	98.1	98.3	90.7	97.5	99.0

**Table 6:** Jetson Nano configuration parameters.

Category	Configuration Parameters
GPU	NVIDIA Maxwell architecture, 128 CUDA cores
CPU	Quad-core ARM Cortex-A57 @ 1.43 GHz
Video Memory	4 GB LPDDR4 (64-bit wide)
Storage	MicroSD card slot (Supports UHS-1 and higher speed cards)
Network	Gigabit Ethernet (RJ45) supports USB Wi-Fi/Bluetooth module expansion
Dimensions	100 × 80 × 29 mm
Power Consumption	5 W (default mode) to 10 W (high-performance mode)

**Table 7:** Comparison of inference time before and after model lightweighting.

Device	Models	Average Inference Time (ms)	Inference Speed (FPS)
Jetson Nano	YOLOv8	137 ms	7.29 FPS
Jetson Nano	PKD-YOLOv8	85 ms	11.76 FPS

## Data Availability

The data used to support the findings of this study are available from the corresponding author upon request.

## Abbreviations

The following abbreviations are used in this manuscript:

PKD	Pruning-Knowledge Distillation
KD	Knowledge Distillation
MGD	Masked Generative Distillation
Cross KD	Cross-modality Knowledge Distillation
CWD	Channel-wise Distillation
FitNet	FITting a Student Network using hints from a Teacher Network
LMGD	Logit-Masked Generative Distillation
BN	Batch Normalization
